# The prevalence of childhood asthma in China: a systematic review

**DOI:** 10.1186/1471-2458-12-860

**Published:** 2012-10-10

**Authors:** Yangzong Yangzong, Zumin Shi, Per Nafstad, Lise Lund Håheim, Ouzhu Luobu, Espen Bjertness

**Affiliations:** 1 Institute of Health and Society, University of Oslo, P.O.Box 1130 Blindern, Oslo 0318, Norway; 2 Tibet University Medical College, Tibet Autonomous Region, China; 3 Division of Epidemiology, Norwegian Institute of Public Health, Oslo Norway; 4 Norwegian Knowledge Centre for the Health Service, Oslo Norway; 5 Institute of Basic Medical Sciences, University of Oslo, Oslo Norway; 6 Jiangsu Provincial Centre for Disease Control and Prevention, Nanjing, China; 7 Discipline of Medicine, The University of Adelaide, Adelaide, Australia

**Keywords:** Asthma, Children, Prevalence, Altitude, Systematic review

## Abstract

**Background:**

It is well known that the prevalence of asthma has been reported to increase in many places around the world during the last decades. Therefore, the aim of this study was to identify and review studies of asthma prevalence among children in China and address time trends and regional variation in asthma.

**Methods:**

A systematic literature search was performed using PubMed and China National Knowledge Infrastructure (CNKI) databases. Selected articles had to describe an original study that showed the prevalence of asthma among children aged 0−14 years.

**Results:**

A total of 74 articles met the inclusion criteria. The lifetime prevalence of asthma varied between 1.1% in Lhasa (Tibet) and 11.0% in Hong Kong in studies following the International Study of Asthma and Allergies in Childhood (ISAAC) protocol. The prevalence was 3% or lower in most articles following Chinese diagnostic criteria. One article reported the results from two national surveys and showed that the current average prevalence of asthma for the total study population had increased from 1990 to 2000 (0.9% to 1.5%). The lowest current prevalence was found in Lhasa (0.1% in 1990, 0.5% in 2000).

**Conclusions:**

The prevalence of childhood asthma was generally low, both in studies following the ISAAC and Chinese diagnostic criteria. Assessment of time trends and regional variations in asthma prevalence was difficult due to insufficient data, variation in diagnostic criteria, difference in data collection methods, and uncertainty in prevalence measures. However, the findings from one large study of children from 27 different cities support an increase in current prevalence of childhood asthma from 1990 to 2000. The lowest current prevalence of childhood asthma was found in Tibet.

## Background

It is well known that the prevalence of asthma has been reported to increase in many places around the world during the last decades [1]. The causes of asthma and why asthma seems to have increased is still not well understood. The increase in asthma prevalence has been suggested in some way to be related to western lifestyle factors, as most often increased prevalence rates are reported from westernized countries [2,3]. Further support for the western lifestyle hypothesis would be to show that the development of asthma prevalence follows the same pattern in societies going through a transition from a more traditional to a more modern lifestyle. Such a transition is currently taking place in China at a much higher speed and during a shorter period than in many other countries [4]. It is likely that the speed of this process and how far the process has already gone varies in different parts of China. Geographically, Tibet is one of the highest regions in the world. Local residents consist mainly of native Tibetans who have lived there for a long time. Furthermore, in Tibet there are areas in which traditional lifestyle still dominates while in Lhasa, the main city, the way of living has already started to develop in a more modern direction due to increasing communication with the rest of the world. Hence, assessing regional population based asthma prevalence and time trends in occurrence of asthma in China and especially in Tibet will provide useful background information for future public health planning, and it may also add to the understanding of how living conditions and ethnic background are related to the occurrence of asthma.

The aim of the current review was therefore to identify and assess studies that have reported asthma prevalence among children in China and search for time trends and regional variation with a special focus on studies among Tibetan children.

## Methods

### Identification of studies

A systematic literature search was performed to identify all relevant publications on asthma prevalence among children aged 0−14 years in China and Tibet, published between 1991 and 2011.

An electronic search was undertaken of the following databases: “PubMed” and the “China National Knowledge Infrastructure (CNKI)”.

For studies based on the protocol of the International Study of Asthma and Allergies in Childhood (ISAAC) we used the following search terms in the PubMed database: (KY= *child* or KY= *childhood*) and KY= *asthma* and (KY= *China* or KY= *Chinese*) and KY= *ISAAC* and KY= *prevalence*. In the CNKI database in articles following ISAAC protocol we used Chinese search terms, here translated into English: (KY= *child* or KY= *adolescent*) and (KY= *asthma* or KY= *bronchial asthma*) and FT= *ISAAC*.

For studies following the Chinese diagnostic criteria we used the following Chinese search terms in the CNKI database, here translated into English: (KY= *child* or KY= *adolescent* or TI= *infant* or TI= *child* TI= *adolescent*) and (KY= *asthma* or KY= *bronchial asthma* or TI= *asthma* or TI= *bronchial asthma*) and (KY= *prevalence* or KY= *epidemiology* or TI= *survey*).

The reference lists of relevant articles were reviewed for additional articles. Some authors of included studies were contacted to identify additional publications. The search results were screened by two authors independently. All included articles were read in full-text.

### Inclusion and exclusion criteria

To be included in the review articles had to meet the following criteria:

1) For studies according to the ISAAC study protocol

a) To report prevalence of asthma among children aged 0−14 years.

b) To follow the ISAAC protocol.

2) For studies based on the Chinese diagnostic criteria

a) To report prevalence of asthma among children aged 0−14 years.

b) To follow one of five sets of Chinese diagnostic criteria for asthma that were established by the First Children Respiratory Diseases Conference (FCRDC) in 1987 [5], the National Cooperation Group on Childhood Asthma (NCGCA) of 1993 or 1998 [6,7], and the Branch for Respiratory Diseases of Chinese Medical Association (BRDCMA) of 1997 or 2003 [8,9].

Articles written in English and Chinese were included. If results based on the same data were presented in more than one publication, results from only one publication were included. Studies lacking clear presentation of prevalence or diagnostic criteria were not included.

### Data extraction criteria

A standardized reporting form was used to extract data from each publication. The form included: first author's name, year of publication, place in which the study was conducted, year of data collection, sample size, age range of study subjects, prevalence estimates and response rate.

### Outcome measures

#### Asthma prevalence according to the ISAAC study protocol

ISAAC was founded to maximize the value of epidemiological research into asthma and allergic disease by establishing a standardized methodology and facilitating international collaboration [10]. For studies following the ISAAC protocol, the lifetime prevalence of asthma was based on the answer provided by 12−14 year-old children to the written question “Have you ever had asthma?” and the answer provided by their parents or guardians to the written question “Has your child ever had asthma?” for 6−7 and 9−11 year-old children [11]. ISAAC has also developed a video questionnaire for assessing asthma symptoms prevalence. The video questionnaire was completed by participants while watching five sequences of persons with different asthma symptoms [12]. Results from assessing the prevalence of asthma symptoms by the video questionnaire are presented when available. ‘Current asthma’ was defined as having asthma with symptoms in the last 12 months [11].

#### Asthma prevalence based on the Chinese diagnostic criteria

For the studies that followed Chinese diagnostic criteria, asthma had to be diagnosed by a physician according to the asthma diagnostic criteria established by FCRDC, NCGCA and BRDCMA between 1987 and 2003. These five sets of diagnostic criteria are similar and prescribe that diagnosis of asthma for children less than three years old had to include the experience of three or more outbreaks of wheezing, and confirmed whistling sounds from the lungs heard by the doctor during examination or documented from medical records. In addition, the diagnosis of asthma for children aged three or older had to meet the criteria mentioned above, and the children had to have experienced a distinct curative effect after treatment with bronchodilators. ‘Current asthma’ was defined as having asthma with symptoms during the last two years [13].

## Results

### Study characteristics

**Figure 1:**
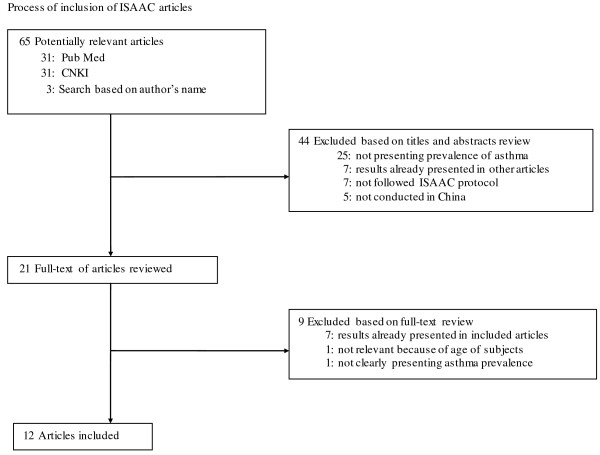
Process of inclusion of ISAAC articles.

We identified 87 potentially relevant articles mentioning ISAAC (Figure 1). From these articles, we excluded 60 articles after reviewing titles and abstracts and excluded 15 more after a full review of the articles. Thus, in this review we included prevalence estimates of asthma from 12 articles (10 in English and 2 in Chinese) [14-25].

**Figure 2:**
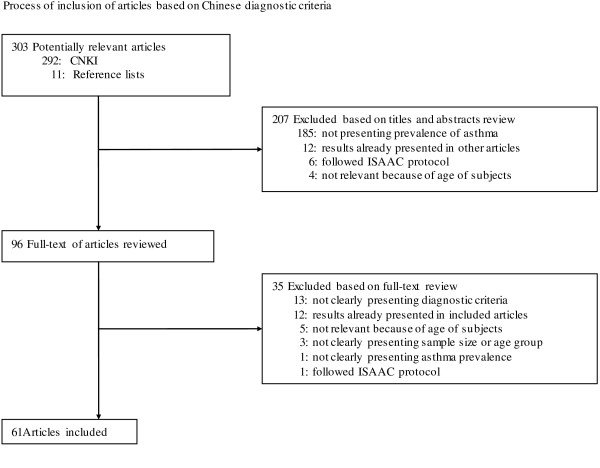
Process of inclusion of articles based on Chinese diagnostic criteria.

We identified 394 potentially relevant articles in Chinese (Figure 2). Frome these articles, 282 were excluded after reviewing titles and abstracts. Consequently a total of 112 articles were reviewed in full-text from which 50 articles were excluded, leaving 62 articles in the final review list [13,26-86].

### Participant characteristics

In seven articles following the ISAAC protocol, children were 12−14 years of age and the questionnaire was completed by the children themselves. In the remaining five articles, the children were aged 6−7 and 9−11 years and the questionnaire was completed by their parents or guardians. In six articles, results from the video questionnaire were also reported. The response rate was more than 90% in all studies.

In 62 articles which followed the Chinese diagnostic criteria, the response rate was reported to be more than 95% in 30 articles while the other 32 articles did not report a precise response rate. Furthermore, half of the articles did not report age specific prevalence of asthma.

### Asthma prevalence

In the ISAAC articles (Table 1) the lifetime prevalence of asthma varied between 1.1% in Lhasa (Tibet) and 11.0% in Hong Kong. The lifetime prevalence was higher among children in Hong Kong compared with children from other cities like Beijing and Guangzhou both among children aged 9−11 and 12−14 years. In contrast, the lifetime prevalence of asthma among children aged 6−7 years was higher in Beijing compared with Hong Kong and Urumqi. One article from Beijing reported that the lifetime prevalence was higher in an urban compared to a rural area (6.3% *vs*. 1.1%) while lifetime prevalence was lower in an urban than a rural area based on reports from two studies in Tibet (1.1% *vs*. 2.5%). One article reported current prevalence of asthma among 10-year-old children in Hong Kong, Beijing and Guangzhou (3.3%, 2.3% and 2.1%, respectively) [25].

**Table 1:** Lifetime prevalence of asthma by ISAAC written questionnaire

**Author, year**	**Place of data collection**	**Year of data collection**	**Sample size**	**Age group (years)**	**Prevalence (%)**
Leung R [14], 1997	Hong Kong	1994−1995	4665	13−14	11.0
Wong GW [15], 2004	Hong Kong	2002	3321	13−14	10.2
Lau YL [16], 1998	Hong Kong	1995	3618	6−7	7.8
Lee SL [17], 2004	Hong Kong	2001	4448	6−7	7.9
Wong GW [18], 2001	Hong Kong	1997−1998	3110	9−11	7.7
	Beijing		4227		6.4
	Guangzhou		3565		4.4
Chen YZ [19], 1998	Beijing	1994−1995	4167	13−14	6.9
	Guangzhou		3855		3.9
	Urumqi		3207		5.4
	Shanghai		3483		7.1
	Chongqing		4296		7.1
Zhao TB [20], 2000	Beijing	1995−1996	2978	6−7	10.7
	Urumqi		2840		7.6
Ma Y [21], 2009	Beijing	2003−2004	Urban 3531	13−14	6.3
			Rural 3546		1.1
Wang HY [22], 2006	Guangzhou	2001	3516	13−14	4.6
Yangzong [23], 2006	Tingri & Sakya (rural Tibet)	2004	2026	12−14	2.5
Droma Y [24], 2007	Lhasa (urban Tibet)	2001	3196	13−14	1.1

Lifetime prevalences of all five asthma symptoms based on ISAAC video questionnaire, were highest in Hong Kong (Table 2). Tibet had the lowest reported prevalences of asthma symptoms and the prevalences were higher in the rural area of Tingri and Sakya than in an urban area of Lhasa. In Guangzhou the prevalences of asthma symptoms were higher in 2001 than in 1994−1995 for four of the symptoms.

**Table 2:** Lifetime prevalence of asthma symptoms by ISAAC video questionnaire

**Author, year**	**Place of data collection**	**Year of data collection**	**Sample size**	**Age group (years)**	**Prevalence (%)**				
					**wheeze at rest**	**wheeze after exercise**	**night waking with wheeze**	**night waking with cough**	**severe asthma attack**
Leung R [14], 1997	Hong Kong	1994−1995	4665	13−14	13.7	21.5	5.5	29.8	9.7
Chen YZ [19], 1998	Beijing	1994−1995	4167	13−14	5.9	7.7	1.4	13.8	2.7
	Guangzhou		3855		3.2	9.2	1.5	11.7	2.2
	Urumqi		3207		2.4	7.0	0.7	11.5	2.5
	Shanghai		3483		3.4	6.0	2.0	4.2	2.8
	Chongqing		4296		2.5	8.8	1.0	6.8	1.6
Wang HY [22], 2006	Guangzhou	2001	3516	13−14	5.9	14.1	1.9	10.2	2.7
Yangzong [23], 2006	Tingri & Sakya (rural Tibet)	2004	2026	12−14	2.8	4.1	2.2	3.8	1.4
Droma Y [24], 2007	Lhasa (urban Tibet)	2001	3196	13−14	0.3	1.2	0.03	1.4	0.3

**Figure 3:**
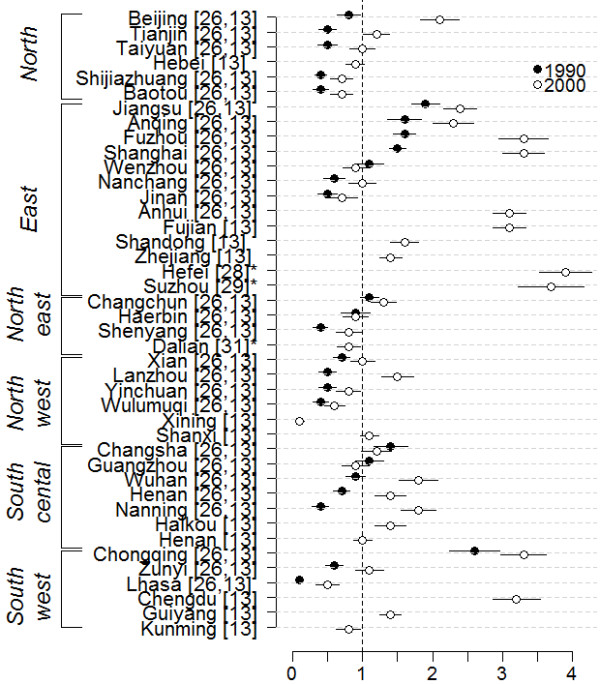
**Prevalence of current asthma (%) in 1990 and 2000. **(* Not part of the national studies. Wulumuqi = Urumqi).

For the asthma prevalence based on the Chinese diagnostic criteria, the current prevalence of childhood asthma from 42 different places in China is shown in Figure 3[13,26,28,29,31]. Most of the results are extracted from two national surveys of a total of 399,193 children aged 0−14 from 1990 and 287,329 in 2000 all living in urban areas [13,26]. The average current prevalence of asthma increased from 0.9% to 1.5% based on estimates from a national survey of children in 27 cities in 1990 and 2000 and statistically significantly increased in 22 out of 27 cities with estimates from both time points [13]. Age stratified analysis showed that the prevalence has increased in most age groups [13]. In addition, we identified three more articles from urban areas carried out in the same period [28,29,31]. The highest current prevalence was found in Chongqing (2.6%) and the lowest in Lhasa (0.1%) in 1990. In 2000, the highest current prevalence was found in Hefei (3.9%) and the lowest in Xining (0.1%). With a few exceptions, cities located in eastern China had higher current prevalences than cities in other parts of the country. There was no clear trend in current prevalence of childhood asthma according to the study populations' living altitude besides that the lowest current prevalence was reported from Lhasa (3,700 meters above sea level) [34] and Xining (2,200 meters above sea level) [35].

In 1990, only three articles reported lifetime prevalence (0.1−0.4%). In 2000, the highest lifetime prevalence was found in Chongqing (4.6%) and the lowest again in Xining (0.3%). Most of these articles reported lifetime prevalences of asthma below 3%. Results from these articles are presented in the Additional file 1: Table S1.

In addition, for the asthma prevalence based on the Chinese diagnostic criteria, we reviewed three more studies conducted in 1991, 2003−2004 and 2007−2008. In Beijing the current prevalence was 0.9%, in Huainan the current prevalence was 3.0% and the lifetime prevalence 4.1%, and in Nanhai the lifetime prevalence was 2% [36-38].

For asthma prevalence based on the Chinese diagnostic criteria, we found 10 articles with uncertainty about whether the reported childhood asthma prevalences expressed current or lifetime prevalences [39-48]. Furthermore, our review included 40 more articles which fulfilled the inclusion criteria. These articles were found difficult to assess due to differences and uncertainties in the presented outcome measures [49-88]. Some results from these 50 articles are presented in the Additional file 2: Table S2.

## Discussion

Lifetime prevalence of childhood asthma based on articles using the ISAAC written questionnaire varied from 1.1 to 11.0% with the lowest prevalence found among Tibetan children. The pattern with lowest prevalence in Tibet was also seen in articles on asthma related symptoms based on the ISAAC video questionnaire and in articles based on the Chinese diagnostic criteria. There were some variation and uncertainties about how asthma prevalences were assessed in some of the articles following the Chinese diagnostic criteria. Even so, most of these articles reported asthma prevalences below 3%. One article presented asthma prevalences for children living in 43 different cities in 2000. Current prevalence varied between 0.1−3.3% and lifetime prevalence varied between 0.3−4.6%. The highest prevalence was found in Chongqing and the lowest in Xining. Lhasa had the second lowest prevalence [26]. Another article presented the current asthma prevalence for large populations of children living in 27 different cities. The average current prevalence estimates were 0.9% in 1990 and 1.5% in 2000 [13].

In this review, the assessment of population-based estimates of asthma prevalence and assessment of regional variation and changes over time turn out to be difficult due to, for instance, differences in diagnostic criteria, data collection methods, sampling of populations and how prevalences are measured. Studies based on the ISAAC protocol relied on self-reports or parental reports of asthma, while this was not sufficient information for the articles following the Chinese diagnostic criteria. In these latter articles physicians had to evaluate the collected information for each potential asthma case and make sure that a set of necessary criteria were fulfilled. The differences described above point towards the need to have reasonably similar disease definitions and data collection methods in order to be able to compare prevalences between studies. Another potential source of uncertainty for the comparisons was the fact that in some articles it was unclear whether lifetime prevalence or current prevalence was presented. The diagnosis of asthma in children younger than three years old was made by clinical criteria, but one could question the reliability of diagnosis of asthma in children at that age. Information on age distribution was often insufficiently presented in studies following Chinese diagnostic criteria, another source of uncertainty when comparing the studies. Taking the above mentioned sources of uncertainty into account we tried to compare articles that seemed to have reasonably comparable outcome definition and data collection methods. For some of the articles this turned out to be more difficult than for others and they were therefore given less attention and results from several articles are only presented in a supplement for completeness.

The five versions of diagnostic criteria for asthma used in articles following the Chinese diagnosis criteria were all quite similar, and we did not consider that these minor differences would substantially distort the comparisons of asthma prevalences between these studies. The size of the population samples were large, especially for the articles based on Chinese diagnostic criteria reducing the problem of random errors.

Secular trend in disease occurrence is difficult to document, especially for diseases like asthma, in which self-report of symptoms has to be a major part of disease ascertainment [89]. It is a common belief that the prevalence of asthma has been increasing in many societies around the world, even if some authors have raised questions about how to adjust for increasing awareness in the populations and among health workers [90-92]. One of the articles in this review was convincingly able to show that there was an average increase in registered cases of current asthma between 1990 and 2000. The study population included a huge sample of children and the data collection methods were comparable for the subsamples of the population. The increase in current asthma prevalence is further supported by the finding of increased occurrence in the majority of the cities and in the age stratified comparisons in the study. The findings support an increased occurrence of asthma within the study period even if one cannot exclude effects of potential changes in awareness and minor changes in diagnostic criteria from 1990 and 2000.

Results from the two national surveys presenting asthma prevalences showed that the prevalences were higher in cities in the eastern part compare to cities in other parts of China. However, the prevalences were low all over China with small absolute differences. Furthermore, the prevalences had more often been assessed in urban than in rural areas, making it difficult to compare the difference in prevalence between urban and rural areas. This was also the case for the ISAAC studies as most of the surveys had been conducted in larger and more modern cities, like Hong Kong and Beijing. Furthermore, the prevalence of asthma in ISAAC studies from China were lower than in ISAAC studies carried out in many other places in the world and especially in developed countries like Austria (32%) [93], United States of America (24.4%) [94], United Kingdom (14.9%) [95] and Singapore (27.4%) [96]. These findings are consistent with the idea that the degree of modernization or westernization is relate to higher prevalence of asthma.

The effect of living altitude was difficult to assess since most studies were conducted in populations living at rather low altitude. One exception was studies from Tibet, which presented some of the lowest prevalence figures regardless of the type of asthma definition used. It is tempting to speculate that factors like the high living altitude, the extreme climate or other living conditions as well as the high child mortality could have contributed to this [97,98]. However, these are speculations, as so far as few studies have been carried out in Tibet to corroborate them. The finding of higher asthma prevalence in rural Tingri and Sakya (4,300 meters above sea level) than in urban Lhasa (3,700 meters above sea level) additionally complicates such speculations. It could only be a by chance finding and both prevalences should be considered as extremely low.

## Conclusions

Assessment of childhood asthma prevalences in China and Tibet, and assessment of regional variation and change over time are difficult due to several uncertainties in the reviewed articles. Asthma prevalence in China was generally low and there were observations supporting the view that asthma prevalence had increased between 1990−2000. The available data gave limited possibility to address regional variation. However, the findings showed that the asthma prevalence in Tibet was in the lowest end of what has been reported elsewhere in China and clearly lower than in most places in the world. Tibet is characterized by extremely high living altitude, extreme climate and high child mortality. The influence of modern lifestyle has only been of minor importance until recently, especially in rural areas. The linking of these conditions to the low asthma prevalence in Tibet is not corroborated yet. It will be interesting to follow future trends in asthma prevalence. Assessing time trends in asthma prevalence is difficult due to the need to rely to some extent on self and parents reporting of symptoms, which may be influenced by increased awareness of asthma in the population as well as among data collectors. Furthermore one needs to ensure that data are optimally collected and comparable with earlier data.

## Competing interest

The authors declare that they have no competing interests.

## Authors' contributions

The authors' responsibilities were as follows Yz: Coordinated the literature review and assessment of review and wrote the manuscript; ZS: contributed in literature review and assessment of review and participated in developing of the manuscript; PN: contributed to the writing of the manuscript; LLH: participated in developing of the manuscript, especially in the methodology part of the manuscript; OL: logistical support for literature review in Tibet; EB: contributed to the writing of the manuscript. All authors read and approved the final manuscript.

## Pre-publication history

The pre-publication history for this paper can be accessed here:

http://www.biomedcentral.com/1471-2458/12/860/prepub

## Supplementary Material

Additional file 1 **Table S1. **Characteristics of included studies--- Prevalence of childhood asthma among 0−14 year-old children in 1990 and 2000. Click here for file

Additional file 2 **Table S2. **Characteristics of included studies--- Prevalence of childhood asthma among 0−14 years old children (uncertain whether it is current or lifetime prevalence). Click here for file
