# Supplementary material for: The prevalence of childhood asthma in China: a systematic review
Source: BMC Public Health. 2012 Oct 10;12:860. doi: 10.1186/1471-2458-12-860 (PMC3524042; doi:10.1186/1471-2458-12-860)
Supplement: Additional file 1 — Table S1. Characteristics of included studies--- Prevalence of childhood asthma among 0−14 year-old children in 1990 and 2000. [file 1471-2458-12-860-S1.doc]

**Supplement** **Table 1 Prevalence of childhood asthma among 0−14 year-old children in 1990 and 2000**

| **Author, year** | **Place of data collection** | **1990** | | | **2000** | | |
| --- | --- | --- | --- | --- | --- | --- | --- |
|  |  | **Sample size** | **Current prevalence (%)** | **Lifetime prevalence (%)** | **Sample size** | **Current prevalence (%)** | **Lifetime prevalence (%)** |
| **Results from two national studies** | | | | | | | |
| NCGCA ,, 2003,2004 | NORTH |  |  |  |  |  |  |
|  | Beijing | 10947 | 0.8 | - | 10163 | 2.1 | 2.7 |
|  | Tianjin | 11593 | 0.5 | - | 12546 | 1.2 | 1.3 |
|  | Taiyuan | 10331 | 0.5 | - | 12104 | 1.0 | 1.3 |
|  | Hebei (shijiazhuang &  handan) | - | - | - | 19832 | 0.9 | 1.1 |
|  | Shijiazhuang | 20222 | 0.4 | - | 9847 | 0.7 | - |
|  | Baotou | 12841 | 0.4 | - | 10914 | 0.7 | 0.8 |
|  | EAST |  |  |  |  |  |  |
|  | Jiangsu (suzhou &  nanjing) | 18200 | 1.9 | - | 16282 | 2.4 | 3.1 |
|  | Anqing | 10635 | 1.6 | - | 10279 | 2.3 | - |
|  | Fuzhou | 25129 | 1.6 | - | 10029 | 3.3 | - |
|  | Shanghai | 43688 | 1.5 | - | 14468 | 3.3 | 4.5 |
|  | Wenzhou | 10009 | 1.1 | - | 9947 | 0.9 | - |
|  | Nanchang | 10000 | 0.6 | - | 10036 | 1.0 | 1.3 |
|  | Jinan | 10761 | 0.5 | - | 5068 | 0.7 | - |
|  | Anhui (hefei & anqing) | - | - | - | 20349 | 3.1 | 4.5 |
|  | Fujian (xiamen & fuzhou) | - | - | - | 21869 | 3.1 | 3.8 |
|  | Shandong (qingdao &  jinan) | - | - | - | 15345 | 1.6 | 1.9 |
|  | Zhejiang (wenzhou &  haining) | - | - | - | 21102 | 1.4 | 2.0 |
|  | NORTHEAST |  |  |  |  |  |  |
|  | Changchun | 22703 | 1.1 | - | 14543 | 1.3 | 1.4 |
|  | Haerbin | 7627 | 0.9 | - | 10046 | 0.9 | 1.2 |
|  | Shenyang | 14669 | 0.4 | - | 9092 | 0.8 | 1.0 |

| **Supplement Table 1** continued | | | | | | | | |
| --- | --- | --- | --- | --- | --- | --- | --- | --- |
| Author, year | Place of data collection | 1990 | | | 2000 | | | |
|  |  | Sample size | Current prevalence (%) | Lifetime prevalence (%) | Sample size | Current prevalence (%) | | Lifetime prevalence (%) |
|  | NORTHWEST |  |  |  |  |  | |  |
|  | Xian | 20002 | 0.7 | - | 12613 | 1.0 | | - |
| **Results from two national studies** | | | | | | | | |
| NCGCA ,, 2003,2004 | Lanzhou | 12069 | 0.5 | - | 11027 | 1.5 | | 1.6 |
|  | Yinchuan | 12961 | 0.5 | - | 10252 | 0.8 | | 1.0 |
|  | Wulumuqi | 12138 | 0.4 | - | 10335 | 0.6 | | 0.7 |
|  | Xining |  |  | - | 10697 | 0.1 | | 0.3 |
|  | Shanxi (xian & baoji) |  |  | - | 23967 | 1.1 | | 1.4 |
|  | SOUTH CENTRAL |  |  |  |  |  | |  |
|  | Changsha | 9009 | 1.4 | - | 10230 | 1.2 | | 1.5 |
|  | Guangzhou | 9979 | 1.1 | - | 8752 | 0.9 | | 1.3 |
|  | Wuhan | 17525 | 0.9 | - | 9137 | 1.8 | | 1.9 |
|  | Henan | 17455 | 0.7 | - | 10572 | 1.4 | | - |
|  | Nanning | 10684 | 0.4 | - | 11238 | 1.8 | | 2.3 |
|  | Haikou | - | - | - | 10421 | 1.4 | | 1.4 |
|  | Henan  (pingdingshan &  zhengzhou & kaifeng) | - | - | - | 21543 | 1.0 | | 1.2 |
|  | SOUTHWEST |  |  |  |  |  | |  |
|  | Chongqing | 7651 | 2.6 | - | 11200 | 3.3 | | 4.6 |
|  | Zunyi | 15105 | 0.6 | - | 9933 | 1.1 | | - |
|  | *Lhasa (urban Tibet)* | 15360 | 0.1 |  | 6676 | 0.5 | | 0.6 |
|  | Chengdu | - | - | - | 10221 | 3.2 | | 4.2 |
|  | Guiyang | - | - | - | 22453 | 1.4 | | 1.8 |
|  | Kunming | - | - | - | 10431 | 0.8 | | 0.9 |
| **Results from local studies** | | | | | | | | |
|  | NORTH |  |  |  |  |  |  | |
| Yan CR , 1994 | Cangzhou (rural area) | 10285 | - | 0.1 | - | - | - | |

| **Supplement Table 1** continued | | | | | | | |
| --- | --- | --- | --- | --- | --- | --- | --- |
| **Author, year** | **Place of data collection** | **1990** | | | **2000** | | |
|  |  | **Sample size** | **Current prevalence (%)** | **Lifetime prevalence (%)** | **Sample size** | **Current prevalence (%)** | **Lifetime prevalence (%)** |
| **Results from local studies** | | | | | | | |
|  | EAST |  |  |  |  |  |  |
| Zheng LL , 2003 | Hefei | - | - | - | 10105 | 3.9 | - |
| Hao CL, 2002 | Suzhou | - | - | - | 6174 | 3.7 | - |
| Wu ZZ , 2003 | Haining | - | - | - | 11155 | - | 2.7 |
|  | NORTHEAST |  |  |  |  |  |  |
| CGCALS , 2002 | Dalian | - | - | - | 10983 | 0.8 | 1.1 |
|  | NORTHWEST |  |  |  |  |  |  |
| CRGCAGP , 1992 | Lanzhou | 11788 | - | 0.4 | - | - | - |
|  | SOUTH CENTRAL |  |  |  |  |  |  |
| Jiang DZ , 1993 | Guangxi (Nanning &  Liuzhou & Baise & Beihai) | 41113 | - | 0.4 | - | - | - |

CGCALS: Cooperation Group of Children's Asthma in Liaoning Shenyang.

CRGCAGP: Cooperative Research Group on Childhood Asthma in Gansu Province.
