# Supplementary material for: The prevalence of childhood asthma in China: a systematic review
Source: BMC Public Health. 2012 Oct 10;12:860. doi: 10.1186/1471-2458-12-860 (PMC3524042; doi:10.1186/1471-2458-12-860)
Supplement: Additional file 2 — Table S2. Characteristics of included studies--- Prevalence of childhood asthma among 0−14 years old children (uncertain whether it is current or lifetime prevalence). [file 1471-2458-12-860-S2.doc]

**Supplement Table 2 Prevalence of childhood asthma among 0−14 years old children (uncertainty whether it is current or lifetime** prevalence)

| **Author, year** | **Place of data collection** | **Year of data collection** | **Sample size** | **Prevalence**  **(%)** |
| --- | --- | --- | --- | --- |
| Han LS , 2002 | Shanghai | 2000 | 1223 | 7.9 |
| Hong JX , 2000 | Xiaoshan | 1997 | 33274 | 3.9 |
| CRGCACHSMC [41], 1995 | Suzhou | 1990 | Urban 6612  Rural 8793 | 3.3  1.4 |
| Wang SY , 2007 | Zaozhuang | 2004 | 12030 | 2.3 |
| Cen SN , 2005 | Foshan | 2000 | 10047 | 2.0 |
| RGPSMC , 1994 | Shanghai | 1990 | 38288 | 1.8 |
| Chen SH [45], 1993 | Jiangsu | 1990 | 60829 | 1.7 |
| Tang PW , 1993 | Zhejiang | 1990 | Urban 21313  Rural 20761 | 0.9  1.2 |
| CRGCAX , 1995 | Urumqi  industrial area  residential area  Aletai  Tulufan | 1990 | 7448  12232  10515  14318 | 0.8  0.1  0.4  0.2 |
| Guo LC , 1992 | Ningxia | 1990 | 30014 | 0.6 |

CRGCACHSMC: Cooperative Research Group on Childhood Asthma at Children's Hospital in Suzhou Medical College.

RRGPSMC: Respiratory Research Group on Paediatrics of the Shanghai Medical Committee.

CRGCAX: Cooperative Research Group on Childhood Asthma in Xinjiang.

Urumqi=Wulumuqi

Table 2 presents results from 10 articles with uncertainty about whether the reported childhood asthma prevalences expressed current or lifetime prevalences. The reported prevalences were lower than 3% except in three cities located in eastern China (Shanghai 7.9%, Xiaoshan 3.9% and Suzhou 3.3%). One article found that the prevalence was somewhat higher in an urban area as compared to a rural area (3.3% *vs.* 1.4%), while in another article the opposite difference was the case (0.9% *vs.* 1.2%).

In addition, our review included 40 more articles which we considered difficult to assess due to differences and uncertainties in the presented outcome measures . Some studies included cases of what was described as “cough variant asthma” and/or “suspected asthma”. Twenty-six studies revealed uncertainty about whether the reported asthma prevalences expressed current or lifetime prevalences. Most articles reported childhood asthma prevalences lower than 3%. The lowest prevalence was found in Lhasa (0.1%) and the highest in Shaoxing (4.6%). Two studies reported that prevalence of asthma was higher in a rural area as compared to an urban area (1.2% *vs.* 0.5% and 0.7% *vs.* 0.5%) .
